# Supplementary material for: Wakefulness can be distinguished from general anesthesia and sleep in flies using a massive library of univariate time series analyses
Source: PLoS Biol. 2025 Jul 10;23(7):e3003217. doi: 10.1371/journal.pbio.3003217 (PMC12244653; doi:10.1371/journal.pbio.3003217)
Supplement: S1 Text — Supplementary figures. (PDF) [file pbio.3003217.s001.pdf]

S1 Text

After the methodological details for the evaluation flies were provided, one systematic difference became apparent between the discovery flies and all the evaluation datasets. In the discovery flies, electrode probes were inserted such that the outermost electrode was positioned just outside the eye of the fly. However, in the evaluation datasets, probes were inserted such that the outermost electrode was positioned within the eye. Because of this difference, we considered that the bipolar re-referenced channels of the evaluation flies would be slightly offset compared to the discovery flies - specifically, that Channel 2 of the evaluation flies (the second deepest channel in the brain) would be better matched with Channel 1 of the discovery flies (and so on for the other channels).

To check whether each channel of the evaluation flies should be matched to take this offset into account, we computed Spearman correlation coefficients among feature values for each pair of one discovery fly and one evaluation fly (giving 637 fly pairs). Stronger correlations when offsetting channels in the evaluation flies would suggest better alignment of discovery and evaluation fly channels through this offset. We first averaged scaled feature values (see Methods of main text) across epochs, per fly and condition. Correlations were then computed for each channel of the discovery flies, with and without offsetting channels in the evaluation flies, using features which were valid across all datasets for the given channel. In the case where channels were offset, we computed correlations between feature values for a given channel of a discovery fly, and the corresponding channel of an evaluation fly (e.g., feature values from Channel 1 of the discovery fly, with feature values from Channel 2 of the evaluation fly), using features which were valid across all datasets for both the discovery fly channel and corresponding evaluation fly channel. Supplementary Figure 1 shows the distribution of Fisher *z*-transformed coefficients across fly pairs and channels, with and without offsetting channels in the evaluation flies.

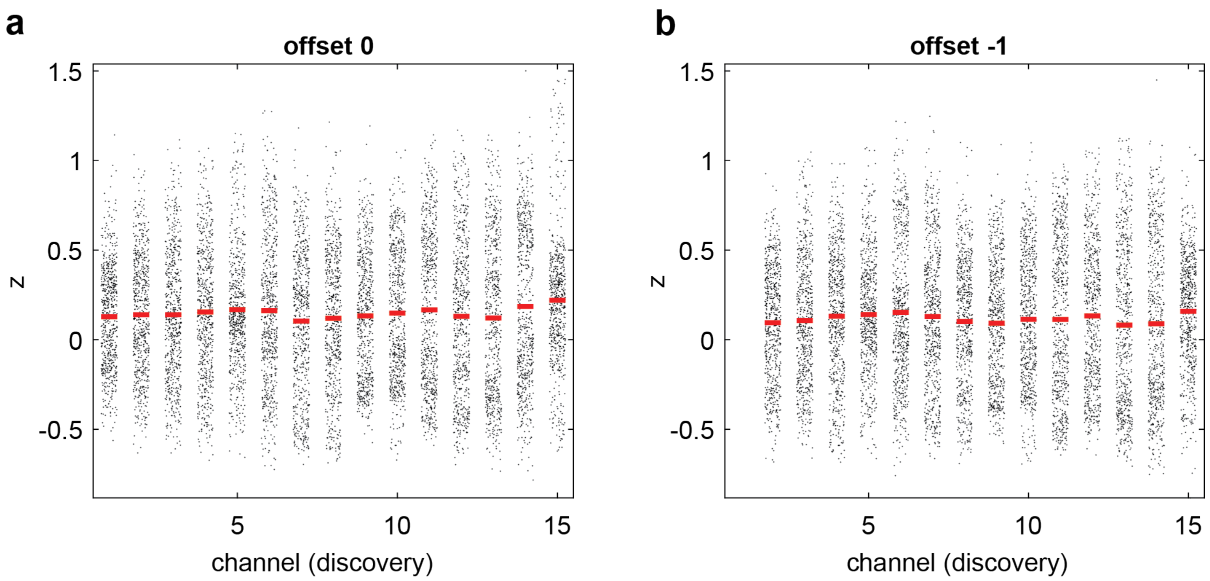

Fig A. Offsetting channels to account for difference in electrode probe depth reduces correlations among *hctsa* feature values.
a) Distributions of correlations (Fisher z-transformed Spearman correlation coefficients) across fly pairs (1 discovery and 1 evaluation fly), at each channel, without offsetting evaluation channels to account for deeper electrode insertion depth. Each point indicates a fly pair. Red lines indicate means across fly pairs. **b)** Distributions of correlations as in **a)**, but when offsetting evaluation channels such that channel 2 of the evaluation flies corresponds to channel 1 of the discovery flies, etc.

To test if correlations were higher when channels were offset in the evaluation flies, we conducted linear mixed effects analysis (LME; [1,2]). LME allows us to account for variability in correlations among the fly pairs and individual channels. Thus, we included random intercepts for fly pair and channel, using the following model (specified in Wilkinson notation [2]):

$$(1)\ z \sim offset + (1|flyPair) + (1|channel)$$

Where z refers to Fisher z-transformed correlation coefficients, *offset*, the fixed effect of interest, refers to whether the evaluation fly channels were offset by one before computing correlations, *flyPair* refers to each pair of one discovery and one evaluation fly, and *channel* refers to each channel in the discovery flies. We employed a likelihood ratio test, comparing the model to a null model without offset as a fixed effect, finding the full model to significantly explain more variance ($\chi^2(1) = 47.56$, $p < .001$). However, the fitted coefficient for offset indicated that correlations were significantly stronger without offsetting the evaluation fly channels ($\beta = -0.03$, $t(18471) = -6.90$, $p < .001$). As such, we concluded that it would be inappropriate to attempt to align the channels of the evaluation flies with those of the discovery flies by offsetting the evaluation flies' channels.

Supplementary Figures

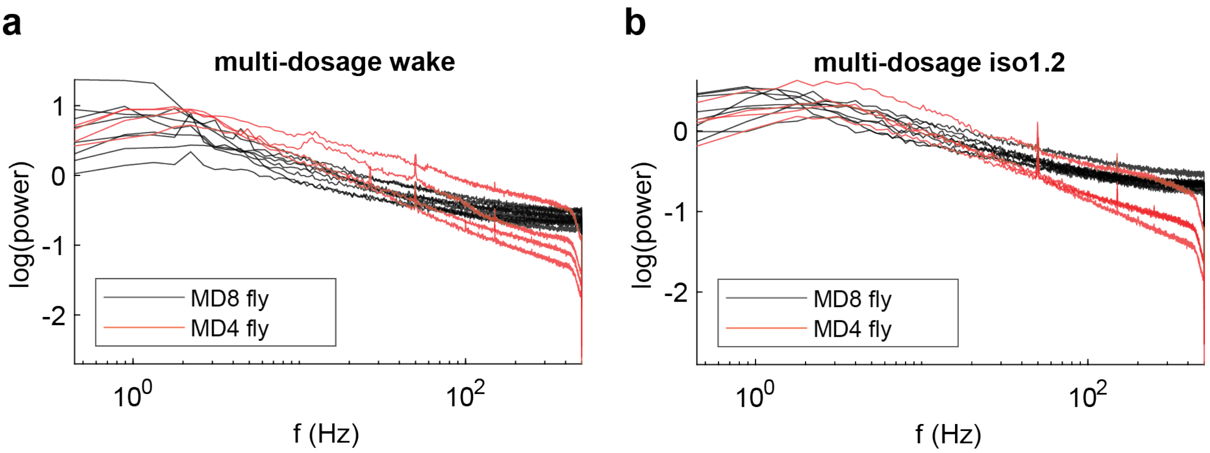

Fig B. Power spectra of MD4 flies compared to MD8 flies.
a) Power spectra for each of the multi-dosage evaluation flies during wakefulness (mean across epochs). Colors indicate MD8 and MD4 flies. **b)** Values as in **a**), but during the 1.2 vol% isoflurane condition.

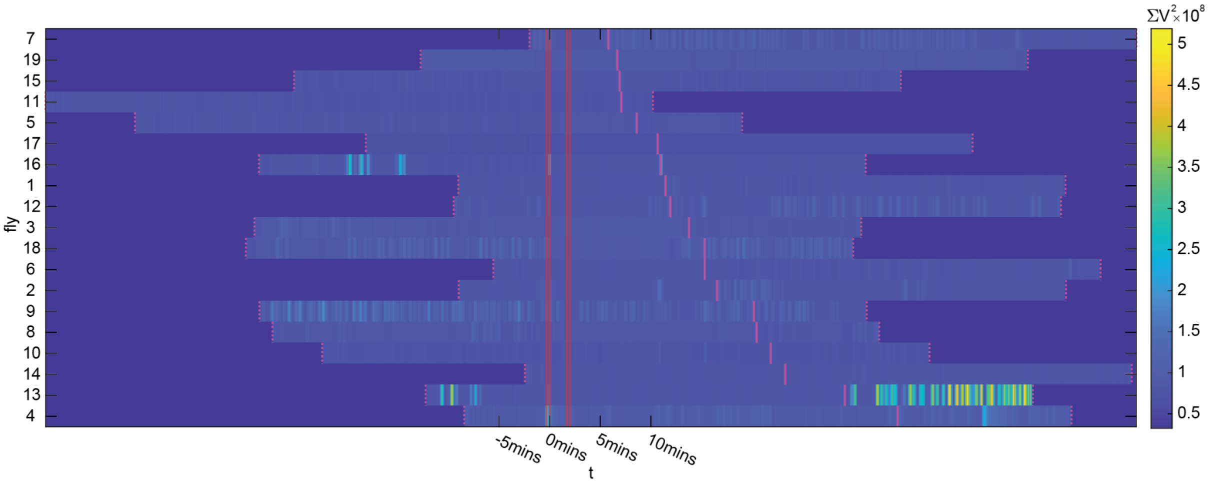

Fig C. Extracted wake and sleep periods in the sleep evaluation flies.
LFPs (voltages squared and smoothed across with an 18 s sliding window) for each of the sleep evaluation flies, averaged across channels. Time-series are aligned by onset of a period of at least 5 minutes where t he fly remained motionless. Vertical, continuous red lines indicate temporal location of extracted 18s segments for the wake and sleep conditions respectively, which we then divided into 8 2.25s epochs. Vertical pink lines indicate when the bout of continuous motionlessness ended for each fly. Dotted red lines indicate start of and end of available recording for each fly.

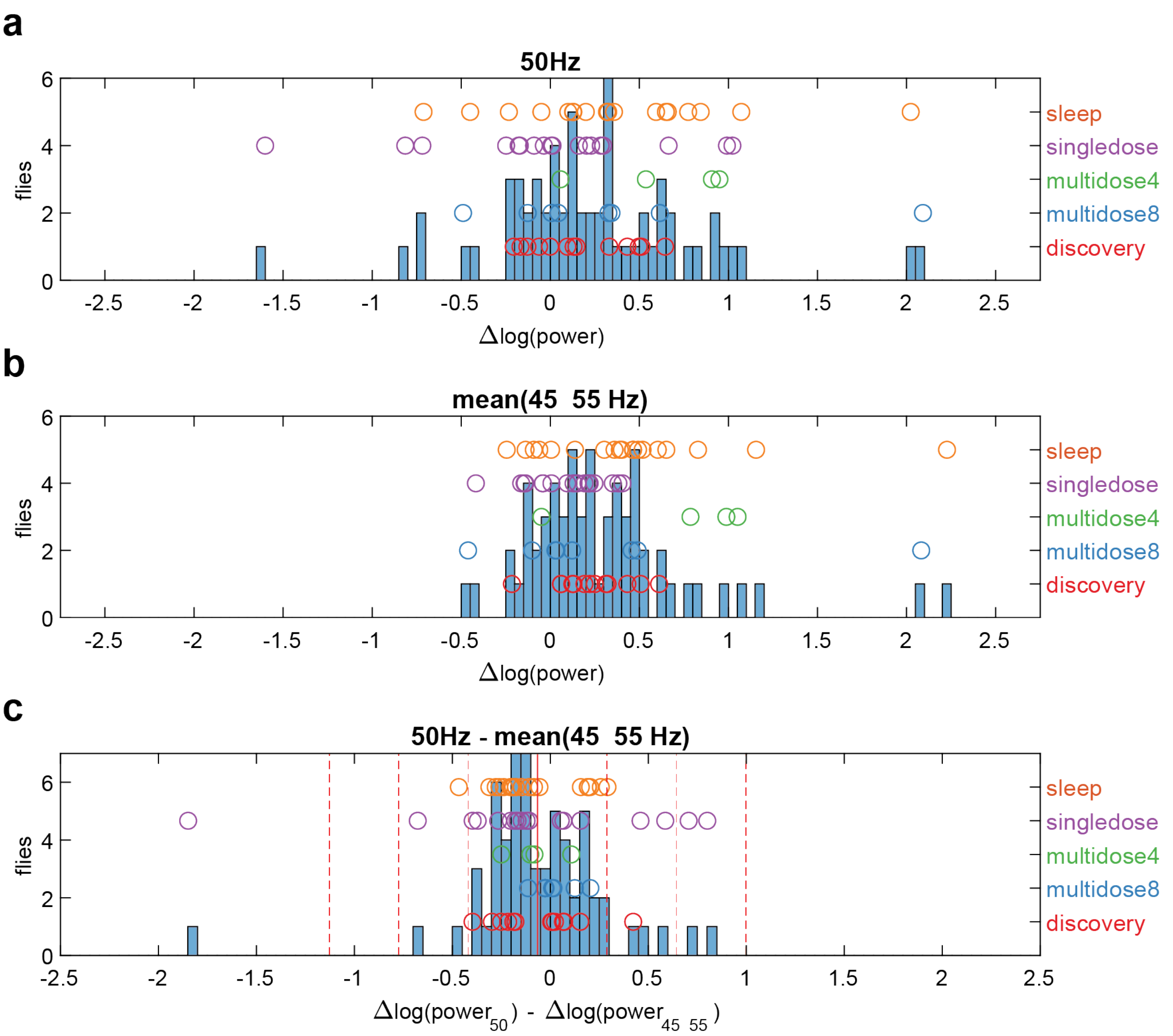

Fig D. Prominence of 50 Hz line noise after pre-processing.
a) Distribution of difference in log power between conditions (wake minus anesthesia/sleep) at 50 Hz (mean across epochs per fly) across flies. Coloured circles indicate mean values for individual flies for each dataset (right-side y-axis). **b)** Distribution of values as in **a**), but for the mean difference in log power at 45 Hz and 55 Hz. **c)** Distribution of differences of values in **a**) (50 Hz) and **b**) (mean of 45 and 55 Hz). Solid and dashed vertical lines indicate mean ± 1, 2, and 3 standard deviations respectively, across flies. For Figure 6d, we excluded the three flies with values outside 2 standard deviations when illustrating the distribution of autocorrelation values among flies.

References

1. Bates D, Mächler M, Bolker B, Walker S. Fitting linear mixed-effects models using lme4. J Stat Softw. 2015;67: 1–48. doi:10.18637/jss.v067.i01

2. Harrison XA, Donaldson L, Correa-Cano ME, Evans J, Fisher DN, Goodwin CE, et al. A brief introduction to mixed effects modelling and multi-model inference in ecology. PeerJ. 2018;6: e4794.
