# Supplementary material for: Wakefulness can be distinguished from general anesthesia and sleep in flies using a massive library of univariate time series analyses
Source: PLoS Biol. 2025 Jul 10;23(7):e3003217. doi: 10.1371/journal.pbio.3003217 (PMC12244653; doi:10.1371/journal.pbio.3003217)
Supplement: Attachment — Submitted filename: Response to Reviewer Comments.pdf [file pbio.3003217.s004.pdf]

We thank the editor and reviewers for their comments. We have revised our manuscript to address the points raised by the reviewers (indented), and provide our specific replies below. We would also like to appreciate the editor for extending the deadline. As you will see below, we have found two more datasets that we can apply our analysis methods to, which will significantly enhance our conclusions with regards to dosage of anesthesia (N=18 additional flies) as well as with loss of consciousness due to sleep (N=19 additional flies). Thanks to the extended deadline, the data curation is complete and we are confident that our novel, blinded, registered analysis will be a suitable contribution to your esteemed journal, *PLoS Biology*. Please see our detailed replies to your and the reviewers' comments below.

REVIEWS:

Academic editor:

> I would invite the authors to specify better **how they will evaluate different effects on different electrodes**. What would it mean if a feature significantly predicts the difference between wakefulness and anesthesia in one channel (either within or across flies), but that same feature does not for another channel?

If a feature significantly predicts wake from anesthesia for some given channel, but not another, then we consider that the feature picks up some particular properties of neural time series which is specific to some brain structure/region. Such properties may reflect highly local neural processes. For example, central brain regions, which include structures such as mushroom bodies, etc. may implement some process which supports higher cognitive processes and/or are involved in regulating the level of consciousness. Meanwhile, this process might not occur in peripheral brain regions. So, features reflecting such a process will be significant in central channels but not peripheral channels. The same logic extends to any channel specificity of feature significance.

This type of functional interpretation with respect to brain regions is possible and has been done in our prior publications (Cohen 2016 eNeuro; Cohen 2018 eNeuro; Munoz 2020 Physical Review Research; Leung 2021 PLOS Comp Bio). As an example, we provide, below, a tentative interpretation of the varying number of significant features across the different channel locations (however, for interpreting specific features, or groups of features, we feel we first need to test for generalization in the evaluation flies). We plan to provide this kind of interpretation in the Stage 2 manuscript, after fully analyzing the blinded data.

"We found channels 4, 7, 8, 11, 12, and 14 to have strong dips in the number of significantly classifying features, compared to neighboring channels. This observation is largely consistent with our prior report (Munoz 2020 Physical Review Research), which analyzed differences between wake and anesthetized states using an epsilon machine method. We extend that isolated finding now to a large number of time-series features.

While these dips may at first seem to suggest that these channel locations are likely unrelated to conscious level, they may also be due to these channels being located at the boundaries of brain structures. For example, Channel 4 is positioned roughly at the boundary between central brain structures (Channels 1-3) and mid-high level visual processing areas (Channels 5-6). Due to this positioning, it is plausible that slight differences

in electrode implantation at individual flies led to recordings at these channels coming from multiple brain regions, depending on the individual fly, and consequently affecting classification performance.

Our results of within-fly effect direction consistency suggest that the above interpretation is indeed the case, for at least Channel 4 where we found consistency to be comparable to neighboring channels. Meanwhile, large differences in the number of significant features both in our classification and within-fly effect direction consistency analyses together are more likely to identify brain regions which are related to level of consciousness or not. For example, Channels 7 and 14, corresponding roughly to the lateral protocerebrum and the lobular plate respectively, have much fewer significant features than surrounding channels, both for classification and consistency. Despite this, the number of significantly consistent features at these channels are not 0 (>100), suggesting that there may be processes from elsewhere in the brain, which are more strongly related to conscious level, which affect these areas. This conjecture is consistent with our earlier report finding widespread integration across the entire fly brain (Leung 2021 PLOS Comp Bio).“

We hope these example paragraphs sufficiently demonstrate the level at which we plan to write our Discussion in the Stage 2 manuscript, regarding channel specificity effects.

> How are the authors going to arbitrate between whether these differences are due to methodological/statistical factors (e.g. different signal-to-noise ratios in the different channels), which may be less interesting, or whether they relate to meaningful differences in the brain structures that are sampled?

In our framework, signal-to-noise ratios (SNR) at each of the channels is not easily defined in a meaningful manner for each individual analytical method (i.e. time-series feature). This is because the concept of SNR is tied strongly with respect to each feature, as well as to the goal of the analysis. For example, for some feature, say related to spectral power, an increase in its value might be considered as a stronger signal. However, that may act as noise for some other feature, for example mutual information. As our overall goal across all the features is to discriminate wakefulness from anesthesia, we think it is appropriate to define SNR as the mean/median of classification performance for a given channel, which is what we do.

Having said this, we have quantified the strength of line noise (at 50 Hz) as an estimate of SNR in a sense of quality control of our recordings. This is implemented as part of our line noise removal pre-processing. As we perform line noise removal, classification performance should not reflect line noise. Further, our signals are high quality local field potentials, recorded from 16 contacts of a single linear silicon probe, and we have noted no SNR issues for our already analyzed data (N=13 discovery and N=2 pilot validation data). So, we expect very little (if any) effect of channel, based on the level of line noise. If we find any noteworthy SNR issue for any part of the unseen validation data, we will report it in Stage 2 manuscript.

With respect to differences in brain structures, please see our (tentative) Discussion paragraphs above. We believe meaningful interpretations can be made by comparing within- and across-fly classification performances across the channels. More coarse-grained comparisons (grouping channels as central/peripheral) can also be made as we have

previously done (Cohen 2016 eNeuro; 2018 eNeuro; Munoz 2020 Physical Review Research; Leung 2021 PLOS Comp Bio).

> If, as anticipated, many neural measures may predict the difference between wakefulness and anesthesia (even for one channel), the authors have to think about an appropriate way to summarize those results in an intuitive manner for the reader.

We agree that if, indeed, many features significantly distinguish wake from anesthesia, then some intuitive summary of significant features will be useful for readers. Given pilot validation results, this may be the case. As an example of such a summary, we show below, for the top 40 performing features (Figure R0.1), how we can group significant features together to interpret general time-series properties which correspond to conscious level. We can adapt and expand this kind of visualisation to accommodate for more features (e.g. by only showing the broad theoretical groupings of features, as indicated by the colour scale in Figure R0.1, and leaving out specific feature names).

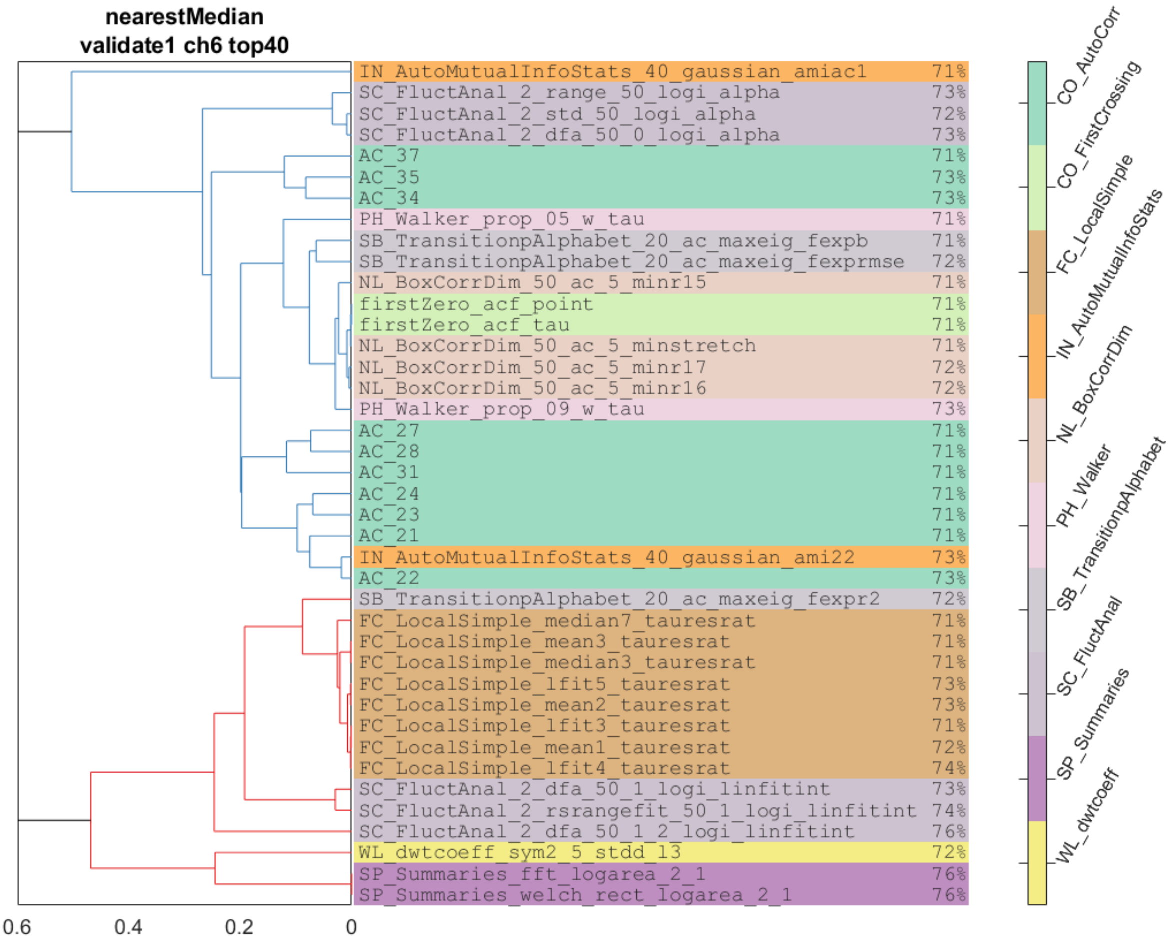

Figure R0.1. Empirical grouping of features with the greatest classification performance (top 40) at Channel 6. Dendrogram is constructed using Spearman correlation distances as linkage distances between every pair of features (computed using all epochs from the discovery and pilot evaluation flies). Dendrogram colours indicate groupings of features, using a threshold of 70% of the maximum linkage. Individual feature names are listed to the right of the dendrogram, along with their classification performance in the pilot evaluation

flies. Colors indicate the broad theoretical category from which individual features belong (descriptions of each category can be found here - https://hctsa-users.gitbook.io/hctsa-manual/list-of-included-code-files). The blue cluster consists of features which are sensitive to the correlation length of time series. Meanwhile, the red cluster consists of features relating both to autocorrelation patterns and proportion of low-frequency power.

We note that it is also possible that, after extending to the full set of validation flies, very few features will remain significant. As the most appropriate way of summarizing the results will depend on the nature of the results, we prefer not to lock in a particular method of summarizing the results at this stage. We have extensively used hctsa in the past (Decat 2021 bioRxiv (accepted by Sleep Medicine, June 2022); Fulcher 2013 Journal of the Royal Society Interface; Fulcher 2017 Cell Systems), and will utilize the tools already available in hctsa as well as potentially other tools to provide the most accessible summary to the reader.

> How are the authors planning to do so? Are there specific “types” of measures that are specifically informative?

We see two potential ways to summarize “types” of informative features from hctsa. The first involves conceptual grouping of features. hctsa groups features with “master operations”, such that features with similar/same theory/mathematical operations are grouped together. One example of this is `SP_Summaries_welch_wmax_<P>`, which computes spectral edge frequency for a particular percentage of total power, `P`. *hctsa* computes separate features for `P` = 5%, 10%, 25%, … 95%.

The second way to summarize “types” of informative features is using a data-driven approach, whereby features are grouped together based on their similarities in the data. For example, features which correlate strongly across all flies, conditions, and epochs, can be grouped together for purposes of interpretation. In Figure R0.1 above, we show an example of grouping features both conceptually (using the color scale) and using a data-driven approach (using the dendrogram).

Recently we have undertaken such a grouping in order to visualize the relationships among many (>300) bivariate measures (Figure 2 in Cliff, Lizier, Tsuchiya, Fulcher 2022 bioRxiv; shown below) for a given dataset, and intend to provide a visualization in a similar vein in order to provide an accessible summary of the broad types of significant features.

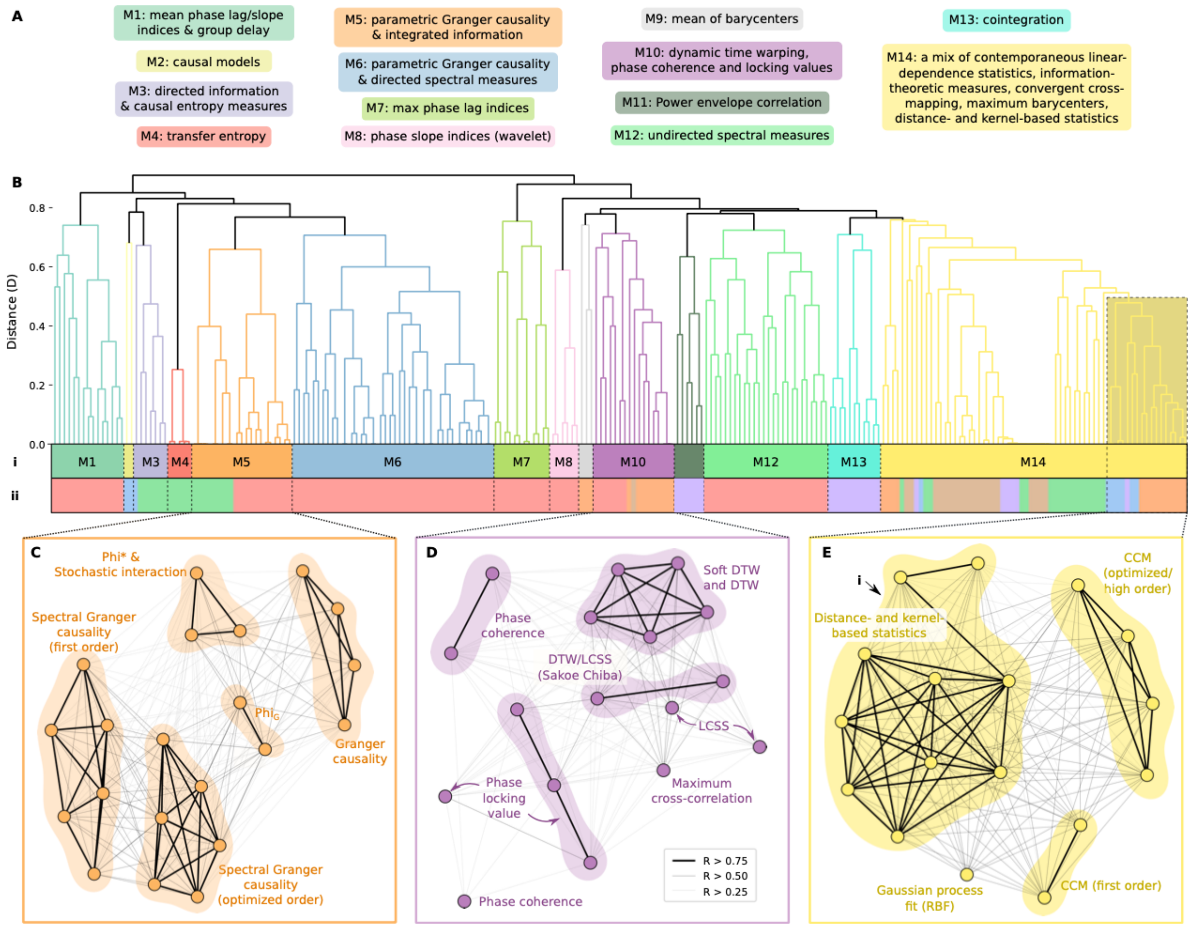

Figure 2 from Cliff et al. A) shows grouping of features based on theoretical groupings. B) shows the empirical correlation structure among features, depicted as a dendrogram. C) visualizes relationships among theoretical groups of features which are empirically clustered together.

> Is there a way to cluster time-series features (see a similar comment by reviewer 4) and link those features back to the existing literature on the known neural processes underlying the change from wakefulness to anesthesia?

As outlined above, we will cluster time-series features based on their similarity across flies, conditions, and epochs. From these clusters, we will identify the overall theme of features within each cluster, and link these to known processes.

Reviewer #1:

> The neural mechanism of anesthesia action remains to be fully elucidated. Using previously recorded LFP signals of flies with/without anesthesia (Cohen et al. 2018 eNeuro), in addition to a newly collected dataset, Leung et al aim to develop a protocol for classifying the depth of anesthesia based on LFP characteristics. Identifying quantitative alterations in LFP signals in the context of anesthesia is quite valuable to a broader neuroscience community beyond the immediate field of anesthetics research. However, based on the authors study design, there are **several conceptual and technical gaps that seem to preclude its potential utility**

to the broader scientific community. At the current stage of this report, this rather specialized study with a debatable conceptual leap in the definition of consciousness seems to be more suitable for a journal dedicated to computational neuroscience.

Major points:

1. It is not clear what (if any) broad biological insight this study by itself will provide. Previous studies by the van Swinderen group establish changes in LFP signals associated with a particular type of anesthetics, isoflurane. However, in this report, the researchers do not propose to compare the time series measures against established differences, nor do they seem to make an attempt to compare a certain subset of interesting features against established measures.

We thank the reviewer for their useful feedback and suggestions. We initially planned to leave these issues for the discussion. Regarding biological insight, the number of significant features at each channel location may point us towards particular brain regions which may be more promising to explore further with regards to linking neural activity/structure to conscious arousal. For example, channels which consistently have many significant features across our datasets (which we have now expanded to include multiple concentrations of isoflurane, and sleep) are more likely to be involved in consciousness than other channels.

Regarding comparison of features to established measures, hctsa pulls its features from various fields of studies. Consequently, some have already been explored as measures of conscious arousal (albeit in human EEG recordings). Given your comment, we have decided to highlight the performances of hctsa features which are related to these previously explored measures of consciousness alongside the top performing features that we have found, in the Results section and in Figures 2c and 3c:

Line 491:

"

For comparison, Figure 2c <panel shown below as Figure R1.1> also shows the performance of hctsa features which are related to previously described indicators of conscious level, but in human electroencephalographic (EEG) recordings. These include features related to spectral power across frequency bands, spectral edge frequencies, and spectral entropy (SP_Summaries), sample entropy (EN_SampEn), permutation entropy (EN_PermEn), single-channel Lempel-Ziv complexity (EN_MS_LZcomplexity), and approximate entropy (ApEn) (Sitt 2014 Brain; Engemann 2018 Brain; Nilsen 2021 PsyArXiv). Surprisingly, the vast majority of these features did not classify wakefulness from anesthesia significantly for both the discovery and pilot evaluation flies.

"

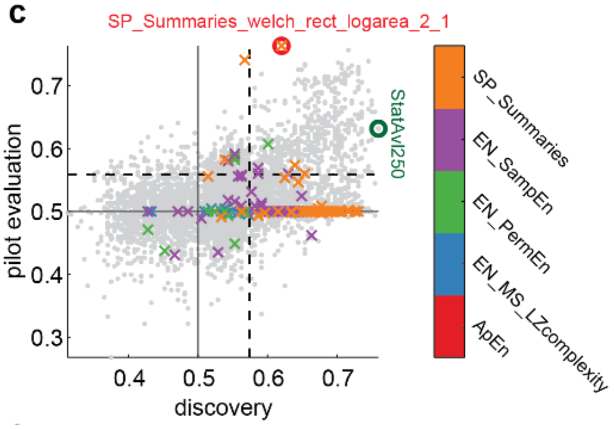

Figure R1.1 (Figure 2c in the manuscript). Correlation of classification performances between the discovery (x-axis) and pilot evaluation flies (y-axis). Each dot represents the classification performance of one of the 6800 features shown in Figure 2a. Solid horizontal and vertical lines indicate chance classification performance (= 0.5). Dashed horizontal and vertical lines indicate the thresholds for statistically significant across-fly classification performance in each set of flies (see Methods). Dots located in the top right quadrant are the features which successfully classified wake from anesthesia across both the discovery and pilot evaluation flies. Coloured x's indicate performance of features related to previously described measures of conscious level, as labeled in the colorbar (features related to spectral power across frequency bands and spectral edge frequency, `SP_Summaries`; sample entropy, `EN_SampEn`; permutation entropy, `EN_PermEn`; Lempel-Ziv complexity, `EN_MS_LZcomplexity`; approximate entropy; `ApEn`).

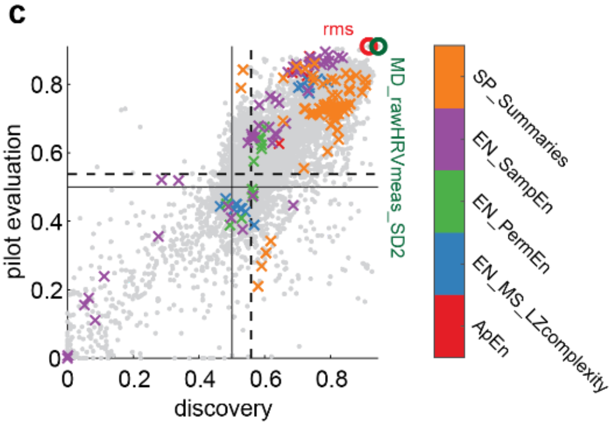

Figure R1.2 (Figure 3c in the manuscript). Correlation of within-fly direction effect consistencies between the discovery and pilot evaluation flies. Format is as described in Figure R1.1.

> Furthermore, as discussed in the manuscript, it is apparent that there will be hundreds of time series features that may be successfully extracted and generalized. What would be the essence of finding these many features?

The essence of finding these many features is in identifying the overall themes of time series properties which are informative as to the level of conscious arousal. We see two potential ways to summarize “types” of informative features from hctsa.

The first involves conceptual grouping of features. hctsa broadly groups features into several themes, such as distribution, correlation, information theory, stationarity, and so on. These broad categories then consist of “master operations”, which implement the computation of a particular measure. Individual features then are master operations which are computed with some specific parameters. One example of this is the individual autocorrelation features in hctsa which are computed at different lags - conceptually, all of these lags are grouped together under one master feature. In the case of these autocorrelation features, the individual features in our dataset are also highly correlated with each other (Figure R1.3).

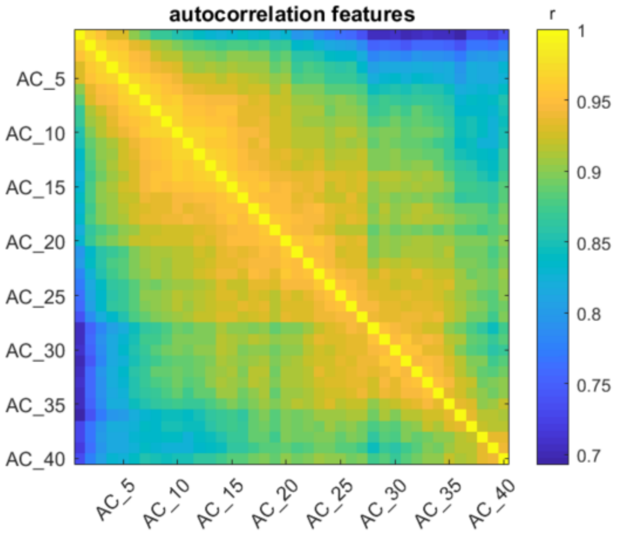

Figure R1.3. Pearson correlation coefficients among autocorrelation features in hctsa at Channel 6. Correlations were computed across all epochs (from both wake and anesthesia) from the discovery flies. Autocorrelation features (AC_1 through to AC_40) are computed with lags of 1 time sample through to 40 time samples.

The second way to summarize “types” of informative features is using a data-driven approach, whereby features are grouped together based on their similarities in the data. For example, features which correlate strongly across all flies, conditions, and epochs, can be grouped together for purposes of interpretation. Figure R1.4 illustrates an example of how we can group features together and also visualize these groups, at one channel location. Given the correlation structure among features in Figure R1.4, we see that features form two broad clusters, one relating to the correlation length of time series (blue) and another corresponding to autocorrelation patterns and low-frequency power (red).

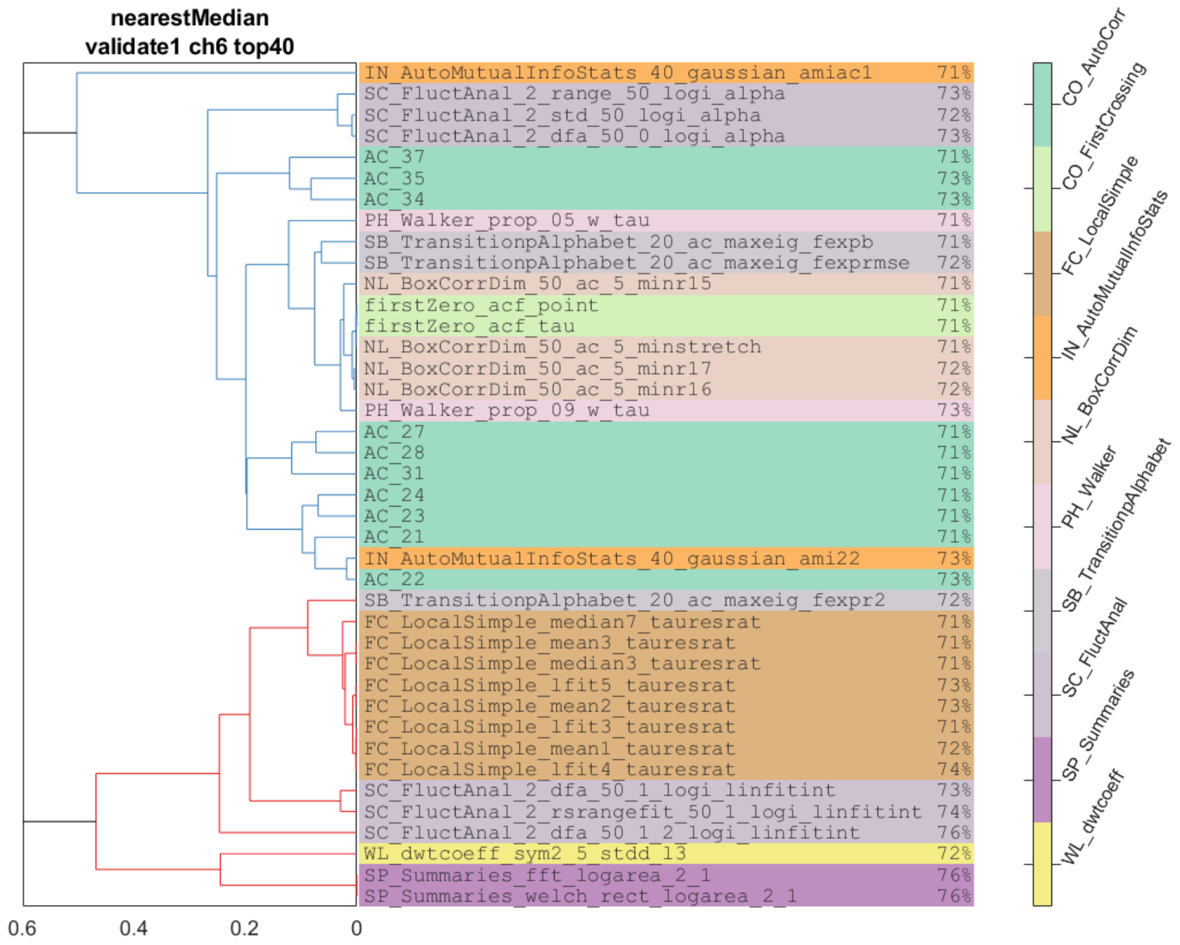

Figure R0.1. Empirical grouping of features with the greatest classification performance (top 40) at Channel 6. Dendrogram is constructed using Spearman correlation distances as linkage distances between every pair of features (computed using all epochs from the discovery and pilot evaluation flies). Dendrogram colors indicate groupings of features, using a threshold of 70% of the maximum linkage. Individual feature names are listed to the right of the dendrogram, along with their classification performance in the pilot evaluation flies. Colors indicate the broad theoretical category from which individual features belong (descriptions of each category can be found here - https://hctsa-users.gitbook.io/hctsa-manual/list-of-included-code-files). The blue cluster consists of features which are sensitive to the correlation length of time series. Meanwhile, the red cluster consists of features relating both to autocorrelation patterns and proportion of low-frequency power.

> More importantly, it is not clear what identification of a particular set of features extracted from data obtained under a single experimental condition will tell us about either biology of anesthesia or a deeper meaning of "consciousness".

We acknowledge this limitation. To address it, we have checked with the data collection team as to the availability of data from other conditions, and confirmed that we will be able to expand our final evaluation dataset to include recordings from flies under multiple concentrations of isoflurane, with recovery from anaesthesia, as well as recordings obtained during sleep. We believe that including these new conditions as in our final evaluation will

allow us to more strongly link identified features to consciousness. We have updated our methods to describe this expanded dataset, as well as the summary table:

Line 248:

"

Multi-dosage evaluation flies

The multi-dosage evaluation dataset consists of 12 isoCJ1 flies, which were administered isoflurane at two concentrations. The 2 pilot evaluation flies described previously and analyzed in Stage 1 of this registered analysis were taken from this dataset (using epochs from wakefulness and one concentration of isoflurane anesthesia). Epochs from this dataset were obtained after an air puff stimulus and consist of 5 possible conditions: 1) wakefulness, 2) isoflurane concentration A, 3) isoflurane concentration B, 4) post-isoflurane (after isoflurane administration, but before flies are fully awake), and 5) recovery (when flies are fully awake and responsive after isoflurane). At time of submission the isoflurane concentrations A and B were not disclosed to the data analysis team.

Single-dosage evaluation flies

The single-dosage evaluation dataset consists of 18 Canton S wild type flies, which were administered isoflurane at a single concentration. Epochs from this dataset were obtained after an air puff stimulus and consist of 4 possible conditions: 1) wakefulness, 2) isoflurane concentration C (which may or may not be equal to isoflurane concentrations A and B in the multi-dosage evaluation flies), 3) post-isoflurane, and 4) recovery.

Sleep evaluation flies

The sleep evaluation dataset consists of 19 Canton S wild-type flies. Recordings were obtained during wakefulness and periods of sleep. Hence, epochs from this dataset consist of 2 possible conditions 1) wakefulness, and 2) sleep.

We intend to update this section when further recording details are revealed to the data analysis team upon in-principle acceptance. We will repeat the methods pertaining to the pilot evaluation flies, described in the following sections, on the final evaluation flies upon in-principle acceptance.

"

2. Dose-response effects. The authors propose to compare a single dose (0.6 % iso) against the no anesthesia condition. However, it is not clear how this dose was selected. Conceivably, a subset of LFP features could emerge at different "levels" of anesthesia. Using this study design, the authors could miss key features related to anesthetic induction, or may generalize effects which are specific to the selected dose. It would be important to explore/analyze the LFP during induction and recovery.

Given the expanded evaluation dataset, we will now analyze recordings obtained under two concentrations of isoflurane, as well as during recovery from isoflurane. Analyzing these conditions may distinguish features which respond to the presence of anesthesia (e.g. by varying greatly between the wake condition and all isoflurane conditions) from features

which correspond more to consciousness (e.g. by varying greatly between only wakefulness and the deeper concentration of isoflurane).

3. Variety of anesthetics. It is utmost important for the authors to demonstrate that the time series features of LFP attributable to "consciousness" (or lack of it) are independent of the types of anesthetics. Otherwise, there will be no ground for the proposal to equate the altered time-series features during anesthesia to a LFP component due to loss of consciousness.

Again, we acknowledge this limitation. While the data collection team does not have datasets involving other anesthetics, they are able to provide recordings obtained during periods of sleep, which we have described above. We believe that including this dataset will allow us to more strongly link identified features to consciousness.

4. The hctsa framework is not well-known to the general biology audience of the Journal. It would be helpful for the authors to better describe the library and to give specific examples of some univariate time-series features and spell out more concrete meaning and implications.

We have expanded our description of hctsa in the Methods section to explicitly describe how time-series features are organized/grouped in the toolbox, and give an explicit example:

Line 288:
"
For a given time series, hctsa extracts a vast set of 7702 univariate time-series features from analysis methods developed in a wide range of scientific disciplines, including nonlinear physics, biomedicine, economics, and neuroscience. *hctsa* broadly groups these features into several general themes, such as distribution, correlation, information theory, stationarity, and so on. Within these themes, features are further grouped into "master operations", which implement the computation of a particular time-series feature. Finally, individual features are master operations computed using specific parameters. An example of this are the features `SP_Summaries_welch_wmax_<P>`, which compute spectral edge frequency at `P`, with `P` = 5%, 10%, 25%, and 95%.
"

5. According to Fulcher et al., 2013, several questions can be raised regarding implementation.
1. Apart from line noise removal, were there any pre-processing/normalization of LFP signals? For several of the hctsa analyses, it would seem that pre-processing was necessary. For example, one would expect that the ST_propsimp function, which measures the proportion of positive vs. negative vs. zero values would be highly sensitive to the DC value of the signal. This measure could be very useful for DC-subtracted data, but meaningless if the DC offset is included.

We did not conduct any other pre-processing or normalization of the LFP signals outside what we described in the methods. We want to minimize selection of pre-processing, as we want to avoid imposing a priori expectations as to which features should be sensitive to level of consciousness (by applying specific pre-processing to support particular features).

Regarding potential features which require DC subtracted data, we do subtract the mean voltage from each epoch (mentioned at Line 278), thus removing DC offset.

2. Many measures include hard-coded parameters, e.g. CO_autocorr uses tau = 1 … 40. Are these parameters values appropriate? One would expect the interpretation of lag would be dependent on sampling rate. A 40-point lag on a 20 kHz signal is very different than that on a 1000 Hz signal.

You are absolutely correct in that interpretation of such features indeed depends on the sampling rate. Any interpretation of features involving hard-coded parameter values would take into account the context of the data, in this case its sampling rate. We will clarify this in Discussion of the Stage 2 manuscript. Interpretation of features across our datasets will be the same as we will pre-process all data with the same sampling rate (1000 Hz).

3. How many of these measures require a signal to be stationary for proper interpretation? Is there evidence that the LFP signals during the anesthesia epochs are stationary?

In general, hctsa features assume stationarity. However, there are also features which serve as measures of stationarity - if there is any difference in stationarity between wakefulness and anesthesia, then these features should distinguish them significantly. An example feature related to stationarity that discriminates wake from anesthesia is `StatAvl250` (`StatAvl250` gives the standard deviation of the mean voltage of non-overlapping windows of 250 timesteps, corresponding to 250 ms in our data), which attained the highest classification accuracy in the discovery flies.

6. Study power & sample size. The authors should justify why a sample size of 13 flies is sufficient for this analysis. Similarly, the test sample size of 12 should be justified. (Both seem somewhat low)

We have now expanded our evaluation flies to include a total of 49 flies (across 2 anesthesia and 1 sleep datasets, as described above). So, including the discovery flies, we will analyze a total of 62 flies. As an exploratory study, with no comparable previous study, we unfortunately do not have a clear way of determining a “stopping rule” to determine the ideal number of flies to analyze. We believe that our expanded sample size, along with our strict blinding and registered report approach addresses concerns related to sample size (along with the other issues brought up in the introduction), and that this study can serve as a starting point for potentially identifying new features to explore as markers of conscious level.

7. False discovery rate. A broader discussion of the Benjamini-Hochberg procedure is warranted as many of the tests in hctsa are clearly not independent to one-another. For example, CO_autocorr, t = 1 and CO_autocorr, t=2 would be expected to yield similar results.

The Benjamini-Hochberg procedure is applicable in the case where the tests are positively dependent, which is likely the case for similar hctsa features (Benjamini 2001 Annals of Statistics; Genovese 2002 NeuroImage). Figure R1.2 shows correlations among autocorrelation features, for example, and shows a positive correlation structure among them. We have updated the methods to state this explicitly:

Line 357:
"
This correction is appropriate when there is a positive dependency among the tests, which is likely to be the case as there are features in hctsa which are expected to give similar results (such as CO_AutoCorr features which compute autocorrelation at different lags)
"

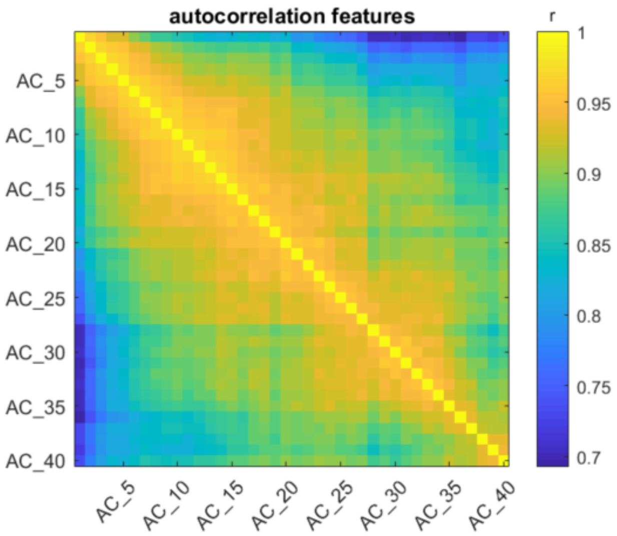

Figure R1.2. Pearson correlation coefficients among autocorrelation features in hctsa at Channel 6. Correlations were computed across all epochs (from both wake and anesthesia) from the discovery flies. Autocorrelation features (AC_1 through to AC_40) are computed with lags of 1 time sample through to 40 time samples.

Reviewer #2: Stage 1 review

The importance of the research question(s).

There are various theories for the neural basis of conciousness which suggest various signatures and mechanisms, however, there is little agreement. I believe the research question posed by the authors, which in short is trying to understand the fundamental mechanisms of conciousness, is important, both from the perspective of scientific understanding but also because it has clinical implications.
The specific research questions, 'What univariate time-series features (from hctsa) can serve as markers of level of consciousness ACROSS individuals?' (and WITHIN), are both important questions which would help us both predict (assess clinical patients for example) or understand mechanisms.

The logic, rationale, and plausibility of the proposed hypotheses (does the manuscript provide a valid rationale for the proposed study, with clearly identified and justified research questions?

The proposed hypotheses (testing each feature at each channel) are logical and rationale. The authors present a data-driven approach to identify time-series that are predictive above chance of conciousness. Until now, most research has been focussed on pre-selected features (either measures of spectral power, or within their own realm of expertise). Taking an indiscrimnate approach removes the feature selection bias. The hypotheses are entirely plausible given that predetermined features have shown some efficacy previously.

The soundness and feasibility of the methodology and analysis pipeline (including statistical power analysis where appropriate). Is the protocol technically sound and planned in a manner that will lead to a meaningful outcome and allow testing of the stated hypotheses?

As made clear by the authors, their methodology introduces a multiple hypothesis problem which the authors will correct for with (i) FDR, (ii) leave one out testing, and (iii) independent evaluation. The protocol would lead to results that a statistically robust. Feasability is clear with the existing hctsa package, pilot results and existing data.

Whether the clarity and degree of methodological detail is sufficient to exactly replicate the proposed experimental procedures and analysis pipeline.

Regarding experimental methodology - it seems clear to me, but I am not an experimentalist and therefore would rely upon other reviewers to check the clarity and detail of the experimental methodology.
The analysis methodology was detailed and clear, and could easily be replicated.

Whether the authors have pre-specified sufficient outcome-neutral tests for ensuring that the results obtained are able to test the stated hypotheses, including positive controls and quality checks.

The authors have clear stated tests that provide outcomes. The authors use an independent classification set, whose importance becomes evident in the pilot results section.

Additional review notes.
The WITHIN fly analysis is quite an interesting approach. Out of curiosity, would WITHIN fly normalisation or WITHIN fly engineering of features not allow the authors to perform a classification task like that used with the ACROSS individual analysis? e.g. normalise values within a fly, instead of across the cohort. A couple of sentences on this might just clear this up for readers.

This can be done, in principle. However, this within-fly normalization would depend on having multiple observations (from the same individual) at different levels of consciousness.

However, such observations from a particular individual may not be available. From the perspective of finding a measure of level of consciousness, then, we would not want to rely on having observations at different levels of consciousness to normalize across.

> There are various methods for batch correction which could improve the generalisability of the ACROSS top features to the independent data set. Is this something the authors have looked into? It would be good to add a sentence or two mentioning this and why it is (or isn't) applicable.

We thank the reviewer for this excellent suggestion! We have added a new analysis, assessing classification performance of features after conducting “batch normalization”, as described below:

Line 340:
“
In both experimental and applied settings, feature values can drift globally across datasets, due to uncontrollable changes in experimental settings or unknown sources of group differences. Some features may be sensitive to such uninteresting drift, which can be corrected automatically. To account for such drift, we report classifier performances both before and after normalizing feature values in the evaluation flies to match the distribution of values in the discovery flies. We normalized each pilot evaluation fly by transforming feature values into z scores, using the mean and standard deviation across all epochs from the other pilot evaluation fly. We then back-transformed the resulting z-scores using the mean and standard deviation across all epochs from all the discovery flies. We will normalize the final evaluation datasets in a similar manner, by transforming feature values into z scores using the mean and standard deviation across all epochs from each set of the evaluation flies and back-transforming using the mean and standard deviation across all epochs from the discovery flies.
“

> Overall, I believe the results will be of interest to the community. Moreover, I think the methodological approach is also useful, both for extending to other analyses/scientific problems and for highlighting the importance of an independent evaluation dataset.

We thank the reviewer for their positive feedback and suggestions.

Reviewer #3, Jacobo Diego Sitt

> I congratulate the authors for this very interesting work. The approach proposed is very interesting and will add an enormous exploration of time-series features for the study of consciousness.

We thank the reviewer for their feedback and suggestions. We hope our replies have addressed the concerns they have brought up.

> My only methodological question refers to the FDR correction for multiple comparisons. Do the authors propose to apply multiple FDR corrections (across

channels) for each feature independently or a unique FDR correction across features x channels? In my view given the number of comparisons, the latter is here the correct approach.

We originally planned to apply FDR corrections across features, for each channel independently. While this gives a more lenient threshold for determining significance of a feature, we expect that many of the features will not remain significant in the final validation flies.

I would like to mention that the authors indicate that prediction generalization using independent datasets is limited in human data. I have to disagree with this statement. It is true that the first studies of biomarkers of consciousness relied on unique datasets to make predictions, but the current standard in the field is to use independent datasets to validate the proposed biomarkers, for example, see reference 14 in your manuscript for EEG or Demertzi Brain 2015 and Sci Adv 2019 for fMRI biomarkers.

Thank you for pointing this out. We have revised the introduction to reflect that while the current standard is currently shifting to validating proposed markers in independent datasets, we can go further with regards to utilizing registered reports and blinding.

Regarding the interpretation of the potential results, I would appreciate it if in the next stage the authors could address in more detail the potential limitations of the weaker form of generalization.

We thank you for your suggestion. We intend to include discussion on the weaker form of generalization, that is, within-fly generalization. In particular, features which have the weaker form of generalization will hold utility in measuring conscious level for individual subjects, when there is some baseline measurement within a subject, such as pre-anesthesia recordings.

In addition, it would be interesting to read the view of the authors of how the proposed approach fits the current discussion of the need for interpretability and explainability for machine learning / biomarkers for putative medical applications.

Again, we thank you for the suggestion. We plan to bring this up in the Discussion. Briefly, as we focus on finding informative features with minimal pre-processing, we can directly interpret identified features or broad themes of significant features. This is in contrast to the approach of more complex machine learning, especially using neural nets, where patterns/markers learnt are obfuscated to the researcher.

Reviewer #4, Tristan Bekinschtein:

The aim to separate, in a data-driven manner, wakefulness (the wake state, either active or passive) from anaesthesia (the anaesthetised state) with neural data is, in principle, useful to understand the underlying signatures of each state and ultimately to further our understanding of consciousness from a neuroscientific perspective. I agree of the strength of using a completely new dataset to test once trained in the

original dataset as the independence would allow to trust the features obtained and imply generalization. I am more worried about the low data amount compared to the level of features and how is that conceptualised. Finally I am asking for further interpretation at the neuroscience level once the data analyses is performed, to conceptually validate the results, as I believe that data science alone cannot define or interpret the result. I comment in more details in the following paragraphs.

I am assuming that the statistical framework, including the permutation and relationship between epochs, electrodes, conditions and flies (test and training sets) are state of the art, I cannot unfortunately be the right expert for that, although I can comment and hope for an explanation as to whether is it correct and valid to evaluate ~700 features in a small set of data (12 flies, 15 electrodes and eight epochs). This is not a critique but a plea for an explanation on how to trust the results with such limited data resources. In particular I don't understand how is FDR applied in each channel for those ~7000 features and whether is gives you a specific cut-off that you trust.

We thank the reviewer for their comments and suggestions. We hope our replies and revisions sufficiently address their concerns.

We have now expanded our evaluation flies to include a total of 49 flies (across 2 anesthesia and 1 sleep datasets). So, including the discovery flies, we will analyze a total of 62 flies (described in the updated summary table, and in the Methods are Line 248). As an exploratory study, with no comparable previous study, we unfortunately do not have a clear way of determining a “stopping rule” to determine the ideal number of flies to analyze. We believe that our expanded sample size, along with our strict blinding and registered report approach addresses concerns related to sample size (along with the other issues brought up in the introduction), and that this study can serve as a starting point for potentially identifying new features to explore as markers of conscious level.

Line 248:

“

Multi-dosage evaluation flies

The multi-dosage evaluation dataset consists of 12 isoCJ1 flies, which were administered isoflurane at two concentrations. The 2 pilot evaluation flies described previously and analyzed in Stage 1 of this registered analysis were taken from this dataset (using epochs from wakefulness and one concentration of isoflurane anesthesia). Epochs from this dataset were obtained after an air puff stimulus and consist of 5 possible conditions: 1) wakefulness, 2) isoflurane concentration A, 3) isoflurane concentration B, 4) post-isoflurane (after isoflurane administration, but before flies are fully awake), and 5) recovery (when flies are fully awake and responsive after isoflurane). At time of submission the isoflurane concentrations A and B were not disclosed to the data analysis team.

Single-dosage evaluation flies

The single-dosage evaluation dataset consists of 18 Canton S wild type flies, which were administered isoflurane at a single concentration. Epochs from this dataset were obtained after an air puff stimulus and consist of 4 possible conditions: 1) wakefulness, 2) isoflurane

concentration C (which may or may not be equal to isoflurane concentrations A and B in the multi-dosage evaluation flies), 3) post-isoflurane, and 4) recovery.

Sleep evaluation flies
The sleep evaluation dataset consists of 19 Canton S wild-type flies. Recordings were obtained during wakefulness and periods of sleep. Hence, epochs from this dataset consist of 2 possible conditions 1) wakefulness, and 2) sleep.

We intend to update this section when further recording details are revealed to the data analysis team upon in-principle acceptance. We will repeat the methods pertaining to the pilot evaluation flies, described in the following sections, on the final evaluation flies upon in-principle acceptance.
“

We intend to apply FDR correction independently for the discovery and evaluation flies. While the specific cut-off for significance may vary between the two datasets, we are interested in whether a feature attains significant performance in both datasets or not.

> If I understand correctly for the second analysis you give a direction of effect based on the median
> "Because the direction of the effect of anesthesia is not necessarily the same across features and channels, we first assigned directionality labels based on the median wakeful and anesthesia values in the 13 discovery flies. For a given feature and channel, we gave a label of 1 if the median wakeful value was greater than for anesthesia, and -1 otherwise. We then multiplied feature values by these labels, flipping the direction of the effect of anesthesia when the median wakeful value is lesser than the median anesthesia value and making the analysis uniform across features and channels. Finally, we report the average proportion across all wakeful epochs and flies."
> I wonder if removing the strictly binary label of 1 and -1 and leaving the normalized difference would give you a direction and also a normalized strength of effect that would shield the results less jerky that the binarization and add more nuanced and strength to the classifiers.

To clarify, we do not binarise feature values. The binary labels 1/-1 describe whether a feature has greater/lower values during wakefulness, compared to anesthesia (based on whether the median wakeful value was greater/lower than the median anesthesia value). The multiplication of the feature values by their 1/-1 label then serves to allow us to analyze all features uniformly, with the assumption that wakeful values are greater than anesthesia values.

> Further to this, it would be good from the beginning to described some of the features, or families of features that separate between conscious states in the original data sets and generalised to the 2 flies in the pilot analyses.

We picked out a couple of specific features to describe at this stage, but after analyzing the evaluation flies we will extend this and describe the themes common to significant features.

We can group features by their theoretical theme or by empirical clustering (as you mention next).

> When having this amount of features it is possible to colour or cluster features according to its characteristics or common mathematical effects, roughly saying what they measure. I see in the manuscript the examples of specific features, but I would like a paragraph on whether there is clustering on features (for figs 2c and 3c) and which of them, as I see in the colourful patterns in the other subfigures (2a and 3a).

We can conduct clustering of features based on the similarity (e.g. correlation) of their values across flies, epochs, and conditions (wake/anesthesia), and provide these for interpretation. As an example, Figure R4.1 shows the empirical grouping of the features with the greatest classification performance (top 40) at Channel 6 in the pilot evaluation flies, along with their theoretical categories. We can use the similar methods for clustering as applied previously to hctsa (such as in Figure 2 of Fulcher 2017 Cell Systems, or Figure 2 of Cliff, Lizer, Tsuchiya, Fulcher 2022 bioRxiv, shown below). However, we note that such clustering methods require some subjective threshold used for determining the clusters.

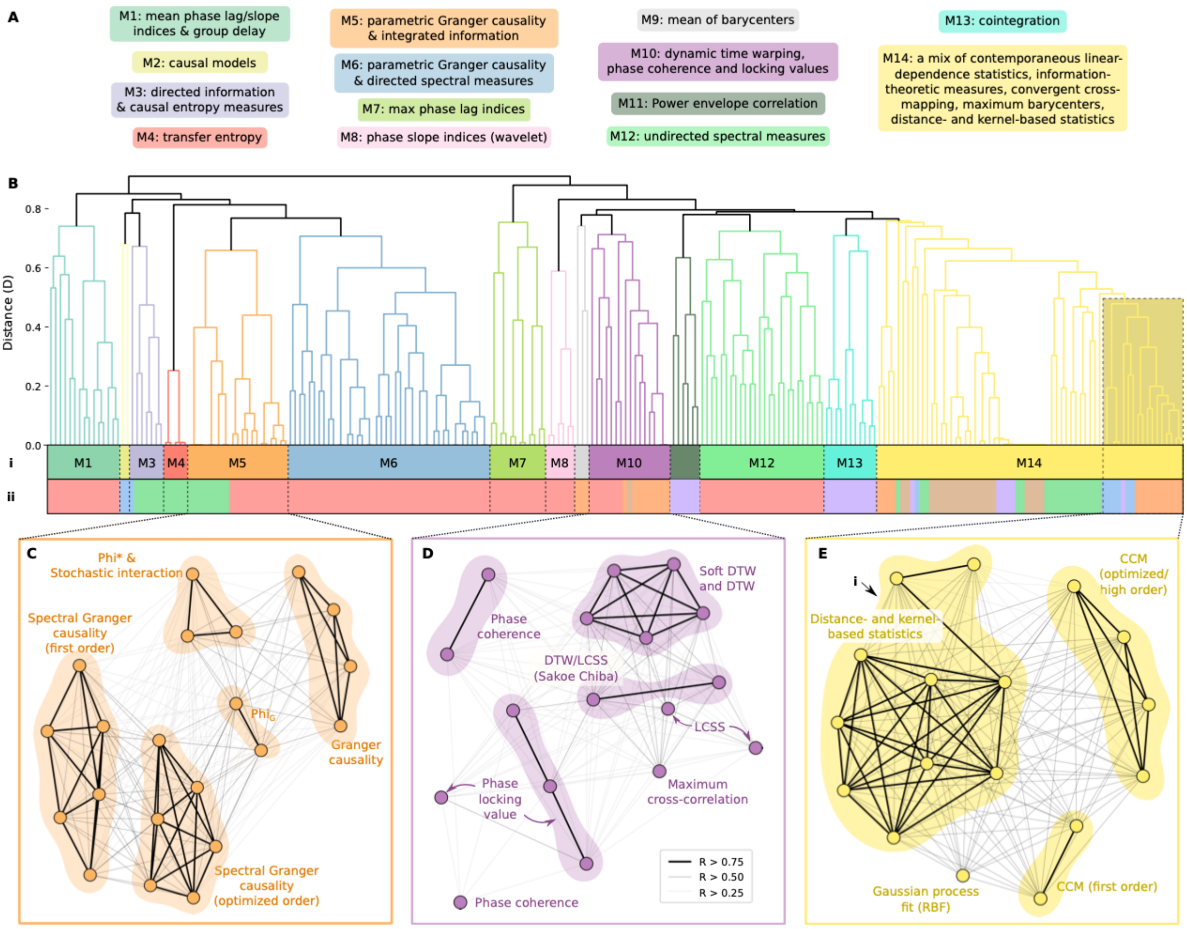

Figure 2 from Cliff et al. A) shows grouping of features based on theoretical groupings. B) shows the empirical correlation structure among features, depicted as a dendrogram. C) visualizes relationships among theoretical groups of features which are empirically clustered together.

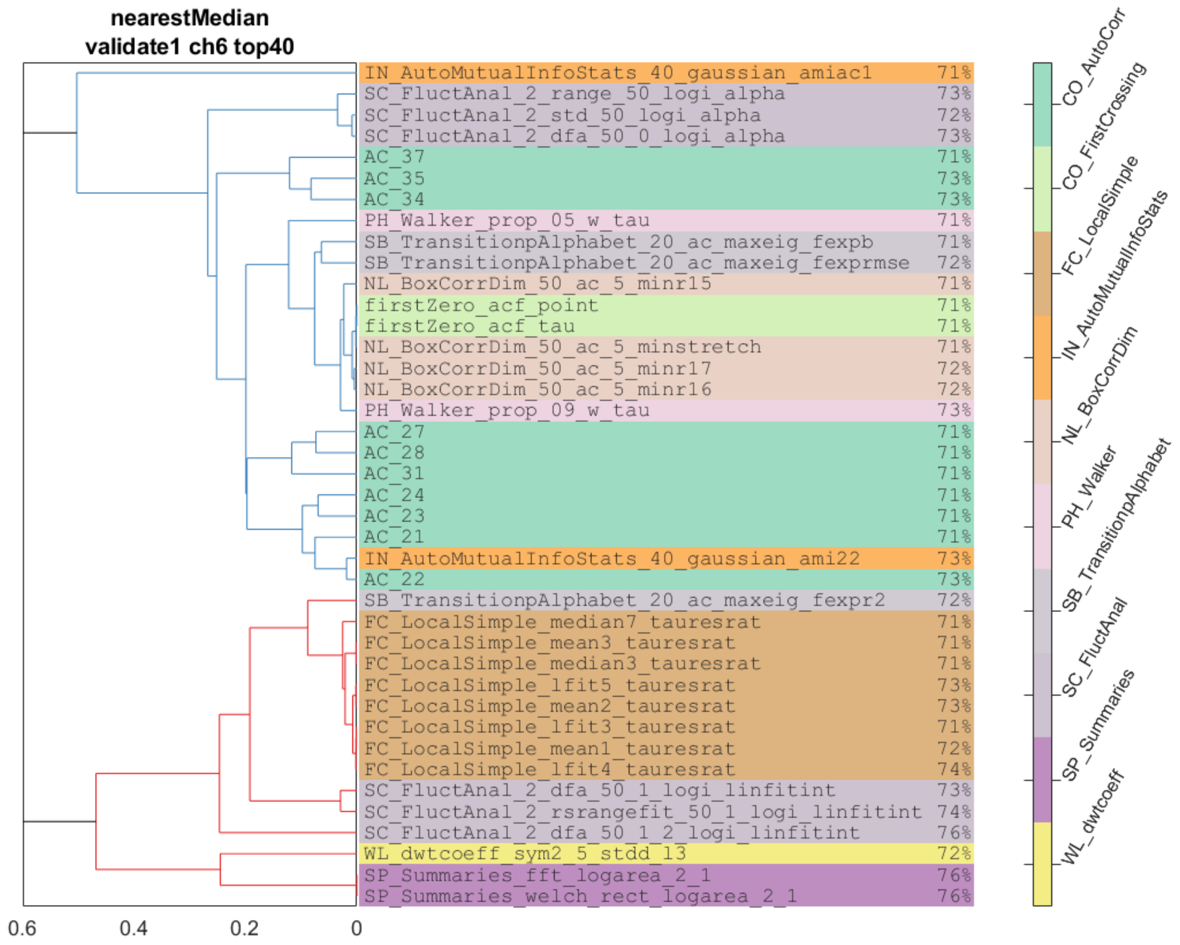

Figure R4.1. Empirical grouping of features with the greatest classification performance (top 40) at Channel 6. Dendrogram is constructed using Spearman correlation distances as linkage distances between every pair of features (computed using all epochs from the discovery and pilot evaluation flies). Dendrogram colors indicate groupings of features, using a threshold of 70% of the maximum linkage. Individual feature names are listed to the right of the dendrogram, along with their classification performance in the pilot evaluation flies. Colors indicate the broad theoretical category from which individual features belong (descriptions of each category can be found here - https://hctsa-users.gitbook.io/hctsa-manual/list-of-included-code-files). The blue cluster consists of features which are sensitive to the correlation length of time series. Meanwhile, the red cluster consists of features relating both to autocorrelation patterns and proportion of low-frequency power.

> Can we trust this 2 features singled and maximal in figure 3c? isn't statistically fishy to have ~95% classification? Depending on the model used there is sometimes penalizing criteria to avoid overfitting classification. I wonder how the authors protect themselves from this, or if they have different interpretation.

We share your skepticism about these features - we think it is unlikely that the high classification performance will extend to the validation flies. Conversely, it would be surprising if they maintained their performance, and worth investigating further in the future. Our protection from overfitting is in utilizing the double blinded registered report approach

that we have presented in this manuscript. We highlighted these high performing features so that readers can potentially track them into the next stage of the registered report and see that specific, “good” features are not guaranteed to generalize to independent datasets.

> This specific result also helps me ask for clusters, and how in that see of blue dots some specific neural features families are together and how that helps interpret the results beyond the simple data-driven aspect. This plea of for us to understand if once the results are given (for the discovery and the pilot) we can go back to neural science and away from data science and start to trust the data output as meaningful for the data we have (LFP) and its conditions (awake and anaesthetised).

We believe our planned clustering above addresses your concern and allows us to interpret the broad themes of significantly performing features in a neuroscientifically meaningful way.

> This aim is clearly stated in the current RR and justified but I am unsure, while reading the introduction, how is this an RR since the preliminary results are already informing. I think there is the need to state clearly which questions and analyses belong to the RR early on in the manuscript by mentioning that are different flies and how will analyses are blinded.

We are glad that you find the pilot results informative already. However, we believe that the critical aspect is whether the promising features identified at this stage generalize to the independent dataset in stage 2. This is particularly important before we make any interpretation of individual features. Given the large number of features (~7000), we do expect some random discriminating features might have performed very well on the discovery (and pilot validation) flies just by chance. Also, as we are interested in finding generalisable features, we need to test them in completely unseen, blinded data as we propose in this registered report. As such, while the current results may already be informing, our aims belong to the entirety of the registered report.

We have revised the introduction to more clearly state the number of flies which are/will be analyzed in each stage:

Line 142:
“
First, we search for features which reliably distinguish wakefulness from isoflurane anesthesia in a discovery dataset (N = 13 discovery flies) and which generalize to a blinded, independent dataset (N = 12 flies with graded levels of anesthesia; N = 18 flies with single level of anesthesia; and N = 19 flies during sleep).
…
At the time of submitting this registered analysis, our early results (on 2 of the evaluation flies) indicate that the performances of many features which have statistically significant performance in classifying wakefulness and anesthesia in the discovery dataset do not generalize to the second, independent, evaluation dataset
“

> A detail in the intro, it might be good to defined what the authors mean by a univariate feature as for example, they ascribed non-univariate states to Lempel-Ziv

Complexity and I would imagine that since it can be calculate on a single electrode, it is not a multivariate feature, or bivariate feature. This might seem a detail but it would be good to hear the classification of the feature the authors have in mind and what are the inclusion or exclusion criteria for them.

We understand that Lempel-Ziv complexity can be computed on a single electrode (in this case, we agree that it should be considered a univariate feature). In the context where we refer to it however (listed among other features which are computed across multiple electrodes), we treat it as multivariate. We have added a clarification that it can be treated both as a univariate (Zhang 2001 IEEE Transactions; Mateos 2018 Cogn Neurodym) or multivariate feature (Schartner 2015 PLOS ONE; Andrillon 2016 Journal of Neuroscience). hctsa implements lempel-ziv complexity as a univariate feature. Figure R4.2 shows how univariate lempel-ziv features in hctsa perform (in blue), among other features.

Line 133:

“

Lempel-Ziv complexity (which can be applied both at the individual channel level as well as across multiple channels)

“

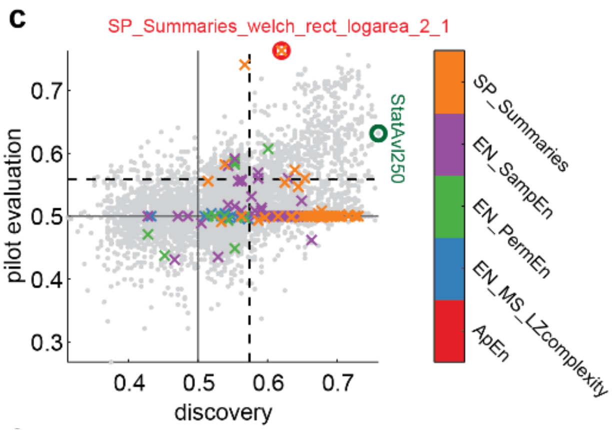

Figure R4.2 (Figure 2c in the manuscript). Correlation of classification performances between the discovery (x-axis) and pilot evaluation flies (y-axis). Each dot represents the classification performance of one of the 6800 features shown in Figure 2a. Solid horizontal and vertical lines indicate chance classification performance (= 0.5). Dashed horizontal and vertical lines indicate the thresholds for statistically significant across-fly classification performance in each set of flies (see Methods). Dots located in the top right quadrant are the features which successfully classified wake from anesthesia across both the discovery and pilot evaluation flies. Coloured x’s indicate performance of features related to previously described measures of conscious level, as labeled in the colorbar (features related to spectral power across frequency bands and spectral edge frequency, `SP_Summaries`; sample entropy, `EN_SampEn`; permutation entropy, `EN_PermEn`; Lempel-Ziv complexity, `EN_MS_LZcomplexity`; approximate entropy; `ApEn`).

In Page 17 "We obtained random-classification distributions for the discovery and pilot evaluation flies by repeatedly classifying discovery or evaluation epochs randomly, with equal probability (as there are 7702 potentially available features in

> hctsa, we repeated this random classification N = 7702 times to build the distribution)." The numbers of features can be corrected to the expected number taking into consideration the limitations described in "Not all of the available time-series features could be extracted successfully from our datasets. For example, the class of features derived from the hctsa function DN_CompareKSFit includes fits of the data to a beta distribution, which assumes values between 0 and 1, an assumption that is not fulfilled by our data and consequently returns missing (NaN) values. To filter out these cases, we excluded any feature which returned NaN across all time series for a given channel in the discovery flies. This reduced the set of features down to an average of 6860 features across the 15 channels (ranging from 6657 to 7004). We further excluded features which returned a constant value across all time series for a given channel in the discovery flies because they are uninformative for classification, reducing the set of features again to on average 6764 features across the 15 channels (ranging from 6560 to 6908)."

In principle, the random-classification distributions will not change as we increase the number of random-classifications used to build them (other than the distribution becoming more finely resolved). So, the choice of repeated random-classification 7702 times is essentially arbitrary. We opted to keep the number of random-classification times constant across channels, and selected 7702 as it corresponds to the number of features in hctsa (as we described in the passage).
