# Supplementary material for: Wakefulness can be distinguished from general anesthesia and sleep in flies using a massive library of univariate time series analyses
Source: PLoS Biol. 2025 Jul 10;23(7):e3003217. doi: 10.1371/journal.pbio.3003217 (PMC12244653; doi:10.1371/journal.pbio.3003217)
Supplement: Attachment — Submitted filename: Response to reviewer comments revision2.pdf [file pbio.3003217.s005.pdf]

We again thank the reviewers for their time and feedback. We have revised the manuscript to address the points raised (indented) and provide specific replies below.

REVIEWS:

Reviewer #1

> We appreciate the authors' genuine effort and the substantial improvements in the manuscript that have made since last review. It is recognized that collecting a robust data set within various readily available sources would enable the authors to draw more convincing conclusions. The inclusion of a wake/sleep data set thus could potentially improve the impact of the paper, provided that the experimental conditions are well defined and the sample size is sufficient for strong inferences. We have a few concerns and suggestions to further strengthen this study:
>
> I. How is sleep defined in the data set?
> Since the recording was performed on mounted flies, sleep indicators based on conventional locomotion activity criteria will be very limited and need additional detectable indicators. This should be spelled out explicitly.

We have added the following to clarify how we defined sleep in the manuscript:

Line 269: While the body of the flies are tethered, their limbs are free to move. We obtained video recordings of the flies to detect and quantify the movement of the flies (Cohen et al 2016 eNeuro). With this movement detection, we defined sleep using the commonly accepted criterion of periods of immobility lasting 5 minutes or longer (Shaw 2000 Science).

> Recent work by the Van Swinderen and Allada Labs (van Alphen et al., 2021Science Advances) has indicated different sleep states in flies correlated with proboscis pumping. Does the data set have enough flies to resolve differences in sleep state? A clear-cut result would be a desirable contribution to the field.

While we are working on this aspect in another project, in this registered report we can potentially analyse and compare two periods of sleep - a light sleep corresponding to the initial 5 minutes of sleep, and what is likely to be a deeper sleep beyond the initial 5 minutes. This 5 minute cutoff corresponds with proboscis extensions associated with deeper sleep (Tainton-Heap 2021 Current Biology; Yap 2017 Nature Communications). This comparison, however, extends beyond the scope of our registered analysis, and we think it may be appropriate to include it as an exploratory analysis rather than as part of our currently proposed analysis plan.

> II. Depth of anesthesia.
> Adding the new data set for a different concentration may reveal the commonality of anesthetic effect and enable a comparison to see the overlap and distinction between sleep (states) and depth of anesthesia. It is still a severe limitation in the scientific inference based on ac single drug to draw conclusions on "anesthesia" effects on LFP features. If appropriately treated, this study may become a significant

contribution to the field. The authors should strive for clearly accessible results based on high-quality data sets from more than one anesthetic compound.

While we agree on the value of analysing multiple anesthetics, this is unfortunately a limitation which we cannot easily get around. Different anesthetics will require different equipment and experimental setups which we currently do not have, such as a different dedicated evaporator. Obtaining multichannel recordings under isoflurane is already an involved setup, and it is currently not feasible for us to develop another setup for another anesthetic for this project.

III. Are the findings of HCTSA parameters sex-specific?
Given the widespread recognition of sex-based differences, the authors should report the sex of the recorded flies, and if possible describe any sex-based differences found in recordings from males and females under wake/sleep/anesthetic states. It is well known that male and female flies display different circadian sleep-wake cycle patterns. An analysis of sex differences would be practically useful to others in the field, and could help identify issues/artifacts originating from electrode positioning (female fly heads are a bit larger and have more facets in their compound eyes).

All the recordings are obtained from adult females. Collecting the multichannel recordings are easier and more reliable in females as they have greater longevity and more likely to survive the recording procedure. Also, as you mention, they are bigger, which also makes it easier to insert an electrode probe. Consequently we cannot address the question of sex-based differences due to electrode positioning. However, we anticipate that any features which we highlight in this project will be subject to further investigation, not just in flies but also in other animals, including humans. In this context, not only sex-based differences, but also species-based differences can be investigated. We have revised the Methods section to clarify that recordings were obtained from female flies (Lines 249, 260, 267).

IV. What is meant by "conscious levels"
A clear operational definition of "consciousness" is required. What is meant by "conscious arousal"? It can be argued that the work is more so an investigation into different sorts of "un-consciousness" and it seems that "brain states" or "arousal states" would be more appropriate terms in describing how sleep and anesthesia induce changes in LFP features associated with different categories of "un-consciousness". The title of the manuscript needs to be appropriately modified to reflect this point.

Thanks for your suggestion. We decided to clarify the title to reflect the fact that we are studying different sorts of “unconsciousness” as you say. The term “level” is a bit technical, thus we changed it into “loss”.

In the field of consciousness research, “consciousness” can be used to mean “level” and “contents” of consciousness (Laureys 2005 Trends in Cognitive Sciences; Boly 2013 Frontiers in Psychology). While level and content are likely to be linked, in this study, we cannot be sure of contents of consciousness in flies without any manipulation about sensory stimuli. What we manipulate is level of consciousness. High level of consciousness means aroused conscious states. We have used the term “conscious arousal” in previous papers

(Munoz 2020 Physical Review Research; Leung 2021 PLOS Comp Bio), and we infer it, as described in the methods, through behaviour (for wakefulness without anesthesia - Line 196: "Flies in the wake condition responded to air puffs by moving their legs and abdomen, but were inert during the anesthesia condition", and for wakefulness in contrast to sleep, through spontaneous limb movements).

Our focus is on distinguishing wakefulness from loss of consciousness, induced by either anesthesia or sleep. Both anesthesia and sleep reduce levels of consciousness. Because of our extended evaluation dataset, we can potentially explore differences between anesthesia and sleep, but we would consider this as an exploratory analysis after our main proposal of finding features which distinguish wakefulness from both anesthesia and sleep. We think that our revised title reflects this primary goal.

> V. Number of independent samples, feature robustness, reproducibility, calibration of analytical tools
> We are still concerned that the number of independent LFP recordings from different flies is too low to draw the intended conclusions. It is strongly encouraged to include additional data sets (new recordings or previously published) to enhance the validity of the conclusions. This is well within the reach of this established group with recognized expertise in this area.
>
> This will help the authors fulfill a major goal: to examine and estimate how robust each (or at least some) of the various features extracted from the signals (time series) in the data set to identify most useful indicators:
> which ones are more tolerant to (or more sensitive to) biological or experiment variability, i.e. which ones stay invariant across different biological samples and/or experimental conditions (such as electrode track precision; biological variation among individual organisms).

With our previous revision, we are now analysing recordings obtained from 47 individual flies. This is comparable to other fly studies which where the order of tens of individual flies are analysed. Regarding experiment variability, each set of evaluation flies (single/multi-dosage anesthesia, and sleep) were obtained in originally independent (unpublished) experiments.

> We were surprised to see that the original description of the multi-electrode approach was not cited (Paulk et al., 2013, J Neurophysiol). The authors should also consider using raw data of recordings from this work to improve the robustness of the analysis. Additionally, Paulk et al have a description of the variability of the electrode insertion tracks. The authors should discuss their HCTSA findings with respect to the variable electrode insertion described in Paulk.

We originally did not cite Paulk 2013 Journal of Neurophysiology as we use a half-brain preparation instead of the whole-brain preparation which is used there. However, we are happy to acknowledge their work as it leads towards the half-brain preparation - we now cite it at Line 184 when describing insertion of the linear probes laterally into the fly's eye. However, we do not see how we can use the recordings from this paper as it does not include recordings obtained during anesthesia. Regarding variable electrode insertion, we

were able to precisely locate them in Paulk 2013 Journal of Neurophysiology with the whole-brain preparation as we knew both the insert and exit points of the electrode at the eyes. With that information, we interpolated the electrode locations inside the brain. As all our recordings in this project come from the half-brain preparation, we cannot use the same strategy to precisely locate the electrodes.

> Instead of a single epoch, analyzing additional epochs or time segments from each individual can better demonstrate certain time-invariant stationary features, uncovering useful time series features and robust parameters.

We indeed do analyse multiple epochs per individual. For the discovery flies, we have analysed 8 epochs each during wakefulness and anesthesia, and we have more epochs available for the evaluation flies (112 epochs each of wakefulness and anesthesia in the pilot evaluation flies).

> Some simple treatments that are more accessible to the general readership of PLoS Biol may be first presented and examined before attempting to interpret overall pictures with a large number of time series features. For example, in Fig R1.3, it could be readily seen whether Autocorrelation features can distinguished sleep, awake and anesthesia states if each of them display best autocorrelation values with distinct ranges of time lags.

We thank you for the suggestion. After finding informative features, we can illustrate the ranges of values that a top-performing feature takes for each of wakefulness, sleep, and anesthesia. We do not wish to do so at this stage while it is still unclear whether any given feature will generalise.

> Many of these points can be empirically determined by the authors with suitable analysis and additional data sets. Comparing the current with the new data sets can also demonstrate the reproducibility and strengthen the conclusions.

Thank you for your comments and suggestions. We hope that our replies and revisions satisfactorily address your concerns.

Reviewer #2:

> The addition of more evaluation flies + a more detailed description of the spatial effects has boosted the proposed analysis.
>
> Personally, the capability to accurately predict/distinguish is what interests me. However, I agree with Reviewer #1, in that whilst I can see what biological insights might come from the analyses, e.g. spatial locations associated with conciousness, I do find it difficult to see how any mechanism could be inferred from some identified time-series feature. For example, if we find a particular time-series feature that is highly predictive, how can this be linked to an underlying neurological mechanism? Of course, this extends more broadly to even traditional neuroscientific features (such as band power), and as such, I don't think its reason to hold this analysis back.

> Interpretation of the top predictive hctsa features and how they could be generated would lead to interesting insights into underlying mechanisms.

It is precisely because traditional neuroscientific features lack such interpretation that we ought to consider other potential features. We hope that looking holistically at the overall types of features which discriminate wakefulness from loss of consciousness will inspire predictions regarding the kinds of neural interactions underlying such features.

> I have no further revision requests.

We thank the reviewer for their time and comments.

Reviewer #3:

> All my comments are properly addressed.

We thank the reviewer for their time and comments.
